# Supplementary material for: Vicarious experiences of touch (mirror touch) in a Chinese sample: Cross-cultural and individual differences
Source: PLoS One. 2022 Nov 18;17(11):e0266246. doi: 10.1371/journal.pone.0266246 (PMC9674153; doi:10.1371/journal.pone.0266246)
Supplement: S3 File — (DOCX) [file pone.0266246.s003.docx]

The data of Study1 is attached in the supporting information. The data of Study 2 and study 3 can be accessed here in http://www.qiujlab.com/news/detail?id=1180
