# Supplementary material for: Vicarious experiences of touch (mirror touch) in a Chinese sample: Cross-cultural and individual differences
Source: PLoS One. 2022 Nov 18;17(11):e0266246. doi: 10.1371/journal.pone.0266246 (PMC9674153; doi:10.1371/journal.pone.0266246)
Supplement: Attachment — Submitted filename: Review for PONE-D-21-29304.pdf [file pone.0266246.s004.pdf]

The Review for PONE-D-21-29304 (Manuscript Title: Vicarious Experiences of Touch (Mirror Touch) in a Chinese Sample: Cross-Cultural and Individual Differences)

This manuscript presents very interesting and sound arguments on the cross-cultural differences regarding the mirror touch synaethesia (MTS) between non-Western (Chinese) and Western (UK) participants. The three experiments are quite persuasively designed and conducted to show that Chinese subjects scored significantly higher than their European counterparts in vicarious experience such as mirror touch. The authors claim that the significant differences in MT-score can be partly attributed to the divergence between Chinese and Western cultures, namely, the interdependent Chinese culture in contrast with European independent culture, and to the tendency of "somatization" in expressing their bodily and affective feelings in Chinese language.

The manuscript is well organized and clearly presented. I expect it will be published to serve as a useful reference for the study of embodied cognition across cultures.

However, there some rooms for improvement with respects of the clarification of some core concepts and more references that need to be included to solidly support the arguments:

(1) The authors fail to mention an essential concept of "interoception" pertaining to "body awareness" and "body perception". Thus, only "exteroception" is included in the discussion of the body awareness and body perception with the crucial roles of "interoception" being overlooked. However, according to Khalsa and her colleagues (2018), interoception is '*the process by which the nervous system senses, interprets, and integrates signals originating from within the body, providing a moment-by-moment mapping of the body's internal landscape across conscious and unconscious levels. Interoceptive signaling has been considered a component process of reflexes, urges, feelings, drives, adaptive responses, and cognitive and emotional experiences, highlighting its contributions to the maintenance of homeostatic functioning, body regulation, and survival*'. That is to say, our personal feelings are of two kinds. First, there are the feelings that come from our bodies. Second, there are affective feelings that relate our moods, dispositions and emotions. And our description of affective feelings in general seems to be associated with the body, in particular, from the perception of the internal organs, muscles, joints and skin etc. (Craig, 2015). Although the manuscript mentioned such terms as "anxiety", "stress" that are associated with "emotions", it failed to giving necessary concerns to "interoception".

(2) the authors should include more information concerning the cultural reasons underlying the MT-score gay between Chinese people and European subjects. It is advisable to cite some references related to the study about the divergence in the notions on body-mind relationship and the conceptualization of emotions across cultures such as "The conceptualization of emotions across cultures: a model based on interoceptive neuroscience" (Zhou et al., 2021), in which, the emotions were proposed to be conceptualized along a continuum with two dimension, namely, bodily transparency and cognitive granularity. For Chinese, emotions tend to be conceptualized with higher bodily transparency but lower cognitive granularity, while for the Western people, emotions are perceived and conceived with lower bodily transparency but higher cognitive granularity. That explains Chinese people's tendency of somatization of emotions and feelings.

(3) The literatures pertaining to the relationship between language and emotion could be cited, for

example, “Language and emotion: Putting words into feelings and feelings into words” (Lindquist et al., 2016) and “The role of language in emotion: predictions from psychological constructionism” (Lindquist et al., 2015). The article “The anger liver, the anxious heart and the melancholy spleen” (Ots, 1990) could be cited in discussing the Chinese view on body-emotion relationship in contrast with Western notions on body-mind-emotion relationship.

(4) There are some minor errors: Western should be capitalized for “W” instead of “western” in the manuscript.
