# Supplementary material for: Vicarious experiences of touch (mirror touch) in a Chinese sample: Cross-cultural and individual differences
Source: PLoS One. 2022 Nov 18;17(11):e0266246. doi: 10.1371/journal.pone.0266246 (PMC9674153; doi:10.1371/journal.pone.0266246)
Supplement: Attachment — Submitted filename: Comments on the manuscript of.docx [file pone.0266246.s006.docx]

**Comments on the manuscript of “vicarious experience of touch across cultures”**

I appreciate the care with which the authors responded to my comments/questions and clarified any potential misunderstanding. The revised manuscript has been strengthened. I am confident that this research paper will become an important reference point for future research in the field of cross-cultural studies on such perceptions as interoception, mirror touch as well as other emotional feelings.
